# Melatonin as a Repurposed Drug for Melanoma Treatment

**DOI:** 10.3390/medsci11010009

**Published:** 2023-01-14

**Authors:** Rachana Pathipaka, Anita Thyagarajan, Ravi P. Sahu

**Affiliations:** Department of Pharmacology and Toxicology, Boonshoft School of Medicine Wright State University, Dayton, OH 45435, USA

**Keywords:** melatonin, drug repurposing, melanoma

## Abstract

Melanoma is the most aggressive type of skin cancer, with a greater risk of metastasis and a higher prevalence and mortality rate. This cancer type has been demonstrated to develop resistance to the known treatment options such as conventional therapeutic agents and targeted therapy that are currently being used as the standard of care. Drug repurposing has been explored as a potential alternative treatment strategy against disease pathophysiologies, including melanoma. To that end, multiple studies have suggested that melatonin produced by the pineal gland possesses anti-proliferative and oncostatic effects in experimental melanoma models. The anticarcinogenic activity of melatonin is attributed to its ability to target a variety of oncogenic signaling pathways, including the MAPK pathways which are involved in regulating the behavior of cancer cells, including cell survival and proliferation. Additionally, preclinical studies have demonstrated that melatonin in combination with chemotherapeutic agents exerts synergistic effects against melanoma. The goal of this review is to highlight the mechanistic insights of melatonin as a monotherapy or combinational therapy for melanoma treatment.

## 1. Introduction

Melanoma is a malignant type of skin cancer that originates from melanocytes, a cell type that is essential for the melanin synthesis required for skin color [1,2,3,4]. The incidence of melanoma has been dramatically increasing worldwide over the last few decades [4,5]. Presently, the prevalence of developing melanoma is 1 in 63 people in the USA [5]. According to statistical estimates by the American Cancer Society, about 99,780 new melanomas (57,180 in men and 42,600 in women) will be diagnosed in 2022 in the USA.

Melanoma, due to its malignant nature, has a poor prognosis, is mainly present in patients with advanced or metastatic disease, and is highly aggressive when compared to other skin cancers such as squamous and basal cell carcinomas [1,2,3,4,5]. The common etiological factors include exposure to ultraviolet radiation, having fair complexions such as red or blond hair, blue or green eyes, and family history of melanoma [4,5,6,7,8]. Ultraviolet (UV) radiations are of three types, namely, UVA, UVB, and UVC, where UVA and UVB can reach and penetrate through the skin layers; however, UVC rays cannot penetrate the ozone layer [6,7]. UVB radiations are more prone to causing melanomas when compared to UVA due to the capacity of UVB to regulate the melanocortin 1 receptor (MC1R) expression, and melanocyte pigmentation. This leads to oxidative changes and endoplasmic reticulum (ER) stress downstream to MC1R, cyclic adenosine monophosphate (cAMP), and inhibition of phosphatidylinositol-3 kinase protein kinase B (PI3K/AKT) signaling [3,6]. This, in turn, alters the melanocyte-inducing transcription factor (MITF), which plays a pivotal role in the differentiation of melanocytes [6]. Alterations in MITF have been shown to cause the rapid proliferation of melanocytes, leading to melanogenesis [3,6,7,9].

## 2. Importance of BRAF Mutations in Melanoma

The cellular and biochemical changes that occur during the pathogenesis of melanoma serve as potential targets for therapeutic interference. For example, initial alterations in melanocytes would result in a benign nevus which remains non-cancerous and is controllable. However, overactivation of the growth regulating mechanisms, such as the mitogen-activated protein kinase (MAPK) signaling pathway, that help support the cell cycle in a homeostatic balance of growth, proliferation, and apoptosis has been shown to result in uncontrollable growth signals, triggered by a single mutation in the MAPK pathway, which leads to cancer [10,11].

Of importance, the v-raf murine sarcoma viral oncogene homolog B1 (BRAF) is a serine/threonine protein kinase that plays a critical role in RAS-RAF-MEK-ERK MAPK signaling. Importantly mutations in the BRAF kinase are the most prevalent driver mutations that lead to the MAPK pathway overactivation [10,11,12]. Within the MAPK pathway, RAF belongs to the family of oncogenic serine-threonine protein kinases. Around half of all metastatic melanoma cases harbor BRAF mutations, particularly, valine (V) for glutamic acid (E) substitution at position 600 (V600E), which accounts for roughly 84.6 percent of all BRAF mutations [13,14]. The substitution of valine (V) for lysine (K) at position 600 (V600K) is a second prevalent amino acid change accounting for 7.7% of BRAF mutations [15,16,17]. While BRAF mutation alone may not contribute to the formation of melanoma, driver mutations in the tumor suppressor genes are frequently required for malignant melanoma progression [12,14,15]. Apart from these, KRAS, NRAS, and P13K/Akt/mTOR mutations also occur in 10% of melanoma cases [12].

## 3. Therapeutic Options for Melanoma

The therapeutic options for melanoma treatment depend upon tumor stages. Primarily, cutaneous melanoma in its early stages with localized lesions can be treated with surgical interventions which have higher recovery options with fewer adverse effects and better quality of life [18]. However, if melanoma progresses to highly aggressive or metastatic states, then treatment strategies vary from chemotherapy to cytokine-based therapies to targeted therapies and immune checkpoint receptor inhibitors [15,17,18]. In chemotherapy, some of the commonly used agents are dacarbazine, temozolomide, lomustine, and vinorelbine, whereas targeted therapy, which is currently considered the first-line treatment option for melanoma, includes BRAF inhibitors such as vemurafenib, dabrafenib, and cobimetinib [18,19].

The BRAF-targeted therapy is used either alone or in combination with the MEK inhibitors such as trametinib, and binimetinib, which are associated with improved survival rates and increased therapeutic effect in patients with BRAF-mutated melanomas [20,21,22]. Although targeted therapies are beneficial, often, tumor cells tend to develop resistance (within several months) to such approaches, resulting in a decreased clinical prognosis of the patients [21,22]. Moreover, such therapeutic options present other limitations, for example, targeted therapy-mediated high response rate is associated with overall short-term therapeutic benefits [23,24,25,26].

Immunotherapies include anti-cytotoxic T lymphocyte antigen-4 (anti-CTLA-4, Ipilimumab), anti-programmed cell death protein 1 (anti-PD-1, nivolumab, and pembrolizumab), and anti-PD-ligand 1 (anti-PD-L1, atezolizumab). These therapies decrease tumor growth and metastasis and are used in combination with other therapeutic regimens, including targeted therapies [23,24,25]. Importantly, immune checkpoint inhibitors have been shown to result in overall higher survival benefits among patients; yet, they are associated with a lower response rate [26,27].

Notably, a few clinical trials reported increased doses of pharmacotherapy to patients, which was associated with adverse effects such as skin rash, keratoacanthoma, hyperkeratosis, headache, arthralgia, pyrexia, and atopic dermatitis, and 7% to 9% cases were shown to develop cardiac abnormalities that include decreased ejection fraction and interstitial lung diseases such as pneumonitis [28]. Therefore, due to these ongoing challenges associated with conventional therapies, including adverse side effects, various adjuvant therapies, including melatonin are being explored as repurposed drugs in cellular and preclinical models because of their anticancer properties in melanoma [29].

## 4. Melatonin as Drug Repurposing

Melatonin (N-acetyl-5-methoxytryptamine) is a chronobiological regulatory hormone produced at night by the pineal gland. It possesses various functions ranging from regulating circadian rhythms to antioxidant, anti-inflammatory, immunomodulatory, and anti-aging properties. Importantly, it also exerts cytoprotective effects in normal cells and triggers apoptotic signals in oncogenic cells [30,31,32,33,34,35,36,37,38]. As melatonin is lipophilic in nature, it can easily penetrate through the cellular membrane to protect intracellular structures such as DNA and mitochondria from oxidative stress induced by free radical generation. Melatonin, due to its pleiotropic actions, has provided the rationale to investigate its anti-proliferative and oncostatic effects and the underlying mechanisms in in vitro and in vivo experimental models of melanoma [31,37,39,40,41,42].

Melatonin is produced by the indole pathway from its precursor serotonin, and then it is metabolized into three different metabolites, 5-hydroxymelatonin, AFMK (N1-acetyl-N2-formyl-5-methoxykynuramine), and 5-methoxytryptamine by the indolic and kynuric pathways. Melatonin and its metabolites mediate their effects through MT1 and MT2 receptors present on the cell membrane [31].

These G-protein coupled receptors are conventionally considered monomers, but they also act as homodimers and heterodimers, which inhibit adenyl cyclase and cAMP. The absorption of linoleic acid is reduced when cAMP synthesis is reduced. The 15-lipoxygenase enzyme converts linoleic acid to 13-hydroxy octadecadienoic acid (13-HODE), which acts as a preliminary energy source for tumor signaling molecules and tumor development pathogenesis.

Importantly, recent evidence indicates that melatonin synthesis and metabolism can also affect tumor microenvironment. In a recent report, Lv and colleagues performed genomic analysis of the melatonergic system within the tumor microenvironment using RNA-seq data from The Cancer Genome Atlas (TCGA) of solid human tumors, including melanoma to determine their clinical relevance [43]. The data demonstrated that melatonin synthesis and its accumulation within the tumor microenvironment negatively correlated with tumor burden as well as mutational burden [43]. Overall, the studies indicated the clinical relevance of the melatonergic system as a promising prognosticator and potential indicator of immunotherapy response.

**Figure 1:**
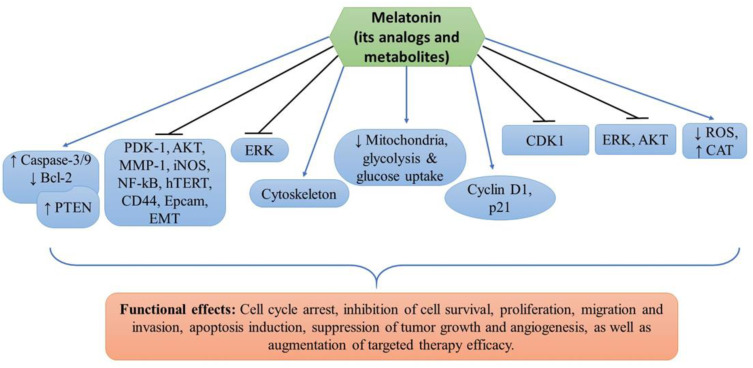
The cellular mechanisms of melatonin and its analogs/metabolites in melanoma prevention. The sign 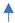 denotes upregulation or increased expression, 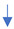 downregulation or decreased expression, and 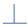 inhibition or suppression.

Melatonin’s antiproliferative and oncostatic actions are thought to be due to its inhibition of linoleic acid absorption [35,44]. Melatonin also activates the apoptosis-targeting proteins p53 and p21. [33,34,35,38,45]. Apart from causing apoptosis, melatonin and its metabolites act as antioxidants forming a free radical scavenger cascade protecting tissues from oxidative damage. Specifically, melatonin protects the skin from UV damage by enhancing the expression of enzymes such as superoxide dismutase (SOD) and glutathione (GSH) peroxidase (GPx), which aid in skin cell protection [44]. The experimental studies determining the effects of melatonin, its analogs, and metabolites are detailed below and summarized in Table 1. The targets of melatonin and its functional effects are highlighted in Figure 1.

## 5. Evidence from Cellular and Preclinical Studies

A study conducted by Bilska and colleagues determined the effects of melatonin, a melatonin precursor serotonin, a kynuric N1-acetyl-N2-formyl-5-methoxykynuramine (AFMK), and indolic pathway metabolites 6-hydroxymelatonin (6(OH)MEL) and 5-methoxytryptamine (5-MT) on mitochondrial function using melanotic (MNT) and amelanotic (A375, G361, Sk-Mel-28) melanoma cell lines [31]. The data demonstrated that treatments with melatonin and its metabolites significantly reduced the proliferation of both melanotic and amelanotic melanoma cell lines in a dose-dependent manner [31]. In addition, melanin content was also found to be significantly decreased at all tested concentrations of melatonin and its metabolites. Interestingly, melatonin, serotonin, AFMK, 6(OH)MEL, and 5-MT treatments not only inhibited mitochondrial and melanosomal stage I-IV development in MNT-1 cells, but also significantly altered mitochondrial oxidative phosphorylation such as reduced ATP, basal and maximal respiration, and increased proton leak and reactive oxygen species (ROS) generation in all melanoma cell lines. These changes resulted in decreased mitochondrial membrane potential, glycolysis, and glucose uptake, leading to the inhibition of melanoma cell proliferation and survival [31].

In another in vitro study, the authors determined the effects of melatonin on cell proliferation and invasion, and defined its mechanisms in different melanoma cell lines such as SK-Mel-19, SK-Mel-147, UACC 62, and normal melanocytes [33]. The investigations showed that melatonin directly affects major cytoskeleton elements by causing G0/G1 phase of cell cycle arrest through alterations in cyclin D1 and p21, which caused decreased cell proliferation and morphological changes in the cell cytoskeleton. In addition, melatonin treatment was shown to disorganize the actin phalloidin and focal adhesions such as paxillin puncta and stressed fiber polarization formation which is high in melanoma cell lines [33]. These data indicate that melatonin possesses anti-proliferative effects on cell mobility during mitosis by affecting the actin microfilaments depolymerization during cytoskeleton remodeling. On the other hand, melatonin effects on skin reconstruction were evaluated using the SK-Mel 147 cell line. The results revealed that melatonin did not affect or only minimally inhibited the migratory ability or invasion of melanoma cells into the dermis, indicating that melatonin could modestly affect the metastatic potential of SK-MEL 147 cells [33].

Along similar lines, Alvarez-Artime and colleagues demonstrated that melatonin treatment caused G2/M cell cycle arrest via decreasing the levels of cyclin-dependent kinase 1 (CDK1), and altered cytoskeleton organization which resulted in decreased proliferation and migration of murine B16-F10 cells [40]. In addition, dose-dependent increased melanin synthesis was observed by melatonin treatment. To determine the mechanisms, the authors found that melatonin inhibited the production of hydrogen peroxide and increased catalase (CAT) enzyme activity, indicating the scavenging of ROS and induction in antioxidant enzyme levels. The in vivo studies demonstrated no differences in the lung metastases with or without melatonin treatment, and that melatonin did not prevent metastasis in this murine model [40].

Guiliana and colleagues determined the mechanisms of antiproliferative activity of new melatonin analogues, namely, UCM 976, UCM 1032, UCM 1033, and UCM 1037 with their binding affinity and intrinsic activity towards MT1 and MT2 receptors [46]. To that end, the authors utilized the NIH3T3 mouse fibroblast cell line stably transfected with human MT1 and MT2 receptors and found that these compounds exhibited affinity towards the MT1 receptor and modest selectivity for the MT2 receptor [46]. The cell viability studies indicated that all melatonin analogues decreased the viability of DX3 and WM-115 melanoma cell lines in a dose- and time-dependent manner, and that maximum cell inhibitory effects were observed with UCM 1033 and UCM 1037 as compared to melatonin alone and vehicle-treated cells. Of all the melatonin analogues, UCM 1037 was selected for apoptosis and necrosis activity in DX-3 and WM-115 cell lines at 24, 48 and 72 h using the flow cytometry technique. The data demonstrated that, compared to DMSO treatment, UCM 1037-treated cells showed pro-apoptotic activity via activating caspase cascade, and specifically at 72 h, the majority of the cells were under late apoptosis and secondary necrosis phases [46]. These studies further confirmed the anti-melanoma activity of melatonin and its analogues.

Importantly, the in vivo experiments were performed using the human DX3 xenograft melanoma model to test the oncostatic effects of UCM 1037 and 1033 and melatonin. Overall, the results showed that UCM 1033 caused a 40% reduction in tumor mass, and UCM 1037 induced a 90% tumor reduction, whereas melatonin only exhibited a modest anti-tumor effect compared with vehicle-treated mice. Mechanistically, melatonin analogues-induced oncostatic and anti-proliferative effects were found to be mediated by the inhibition of phosphorylation of extracellular signal regulated protein kinase (ERK)-MAPK and AKT. Additionally, melatonin also induces pro-apoptotic caspase-3 cleavage resulting in apoptosis [46].

A study by Agil and colleagues investigated the chemopreventive effect of melatonin on the growth of the B16-F10 murine metastatic melanoma model. To that end, C57BL/6 mice were pre-treated orally with or without melatonin for 14 days followed by subcutaneous implantation of melanoma cells and then the treatment continued at the end of the study. The data demonstrated that melatonin treatment significantly reduced tumor size and increased the survival probability of mice, which was correlated with tumor size as compared to the vehicle-treated control mice [47]. Mechanistically, melatonin-induced decreased tumor growth was mediated via increased tumor cell degeneration and necrosis as well as reduced tumor angiogenesis, mitotic index, cell proliferation, and activation of the ERK1/2 signaling pathway. Importantly, while no differences in the mitochondrial complex activities were noted, a significantly increased level of mitochondrial nitrites was found in melatonin-treated mice compared with the control mice. Overall, the findings indicated the potential chemopreventive efficacy of melatonin in the experimental melanoma model [47].

Importantly, the authors also determined the synergistic effects of melatonin in combination with vemurafenib in both the in vitro and in vivo models of melanoma. The cell proliferation was assessed in various BRAF mutant SK-Mel-28, A375, and G36 cell lines, as well as the BRAF wild type A431 cell line. It was observed that the melatonin and vemurafenib combination reduced the cell viability and the colony-forming ability of melanoma cells as compared to the treatments with individual agents [11]. Importantly, the combination treatment was found to decrease the activation/phosphorylation of oncogenic signaling pathways, 3-phosphoinositide-dependent protein kinase-1 (PDK1), and AKT and increase the activation of the tumor suppressive gene, phosphatase and tensin homolog (PTEN) expression, which explains the enhanced sensitivity of vemurafenib and melatonin towards melanoma [11]. Mechanistic studies have shown that melatonin enhanced vemurafenib efficacy in the inhibition of cell migration and invasion of melanoma cells; and decreased the expression of matrix metalloproteinase 1 (MMP-1), vimentin, and β-catenin; and upregulated the expression of E-cadherin, indicating that this combination inhibits epithelial-to-mesenchymal transition (EMT) [11].

Furthermore, this combination treatment was found to induce increased apoptosis mediated via the mitochondrial pathway as measured by increased cleavage of caspase 3 and 9, PARP, decreased expression of Bcl-2, as well as enhanced cytochrome c release as compared to monotherapy. Interestingly, the authors also found that the combination treatment reduced the iNOS expression by inhibiting the nuclear factor kappa B (NF-kB) pathway [11]. Simultaneously, not only significantly suppressed phosphorylation of the inhibitor of nuclear factor kappa-B kinase subunit beta (IKKβ) in melanoma cells was observed, without affecting its overall expression, but also decreased expression level of phosphorylated IκBα, as well as cancer stem cell characteristics, for example, the downregulation of human telomerase reverse transcriptase (hTERT) was noticed. Apart from this, the combination treatment significantly reduced the growth of tumor xenografts in the mouse melanoma model [11]. The results of the protein and immunohistochemistry analyses showed that the combination of vemurafenib and melatonin not only predominantly suppressed the expression of hTERT, inducible nitric oxide synthase (iNOS), p65, CD44, and the epithelial cellular adhesion molecule (Epcam), but also markedly reduced the level of proliferating cell nuclear antigen (PCNA) in tumor xenografts, compared with the single drug treatment. Taken together, these studies indicated the potential of melatonin in enhancing the efficacy of BRAF-targeting agents in melanoma treatment [11].

## 6. Conclusions

Collectively, based on in vitro and in vivo studies, melatonin possesses promising anti-cancer properties via its ability to regulate multiple cell signaling pathways such as MAPK and PI3K/Akt/mTOR. In addition, melatonin has been shown to regulate cytoskeleton remodeling during the mitosis phase of the cell cycle, resulting in the inhibition of melanoma growth. Importantly, melatonin exerts synergistic effects in combination with vemurafenib, resulting in the augmentation of vemurafenib efficacy against melanoma. Considering the beneficial therapeutic window of combination therapy that outweighs monotherapy responses, including overcoming tumor resistance mechanisms, future studies determining the relevance of other pathways [48,49,50] in the therapeutic responses of melatonin could provide novel strategies against melanoma.

## Figures and Tables

**Table 1:** Summary of the in vitro and in vivo studies.

Cell Lines Used	Drug(s)	Findings	Targets	Refs.
Sk-Mel28, A375, G361, A431	Melatonin and vemurafenib	Inhibition of cell proliferation and induction of apoptosis	PDK-1, AKT, PTEN, MMP-1, EMT, NF-kB, hTERT, iNOS, CD44, and Epcam	[11]
A375, G361, Sk-Mel28, MNT-1	Melatonin, serotonin, AFMK, 6(OH)MEL and 5-MT	Inhibition of melanoma cell proliferation and survival	Mitochondria, glycolysis and glucose uptake	[31]
SK-Mel-5, SK-Mel-19, SK-Mel-28, SK-Mel-29, SK-Mel-103, SK-Mel-147, G-361, UACC 62 and normal melanocytes	Melatonin	Cell cycle arrest, inhibition of cell proliferation and invasion, and cytoskeleton remodeling	Cyclin D1 and p21	[33]
B16F10 melanoma cells	Melatonin	Inhibition of cell proliferation, migration, and increased melanin synthesis. G2/M cell cycle arrest, altered cytoskeleton organization, reduced ROS and increased CAT enzyme activity	CDK1	[40]
DX3 and WM 115	Melatonin, UCM976, UCM1032, UCM1033, and UCM 1037	Inhibition of cell viability, decreased tumor growth and induction of apoptosis	AKT and ERK	[46]
B16-F10	Melatonin	Reduced cell proliferation, tumor growth and angiogenesis, and increased mice survival probability	ERK	[47]

## Abbreviations

Ultraviolet, UV; melanocortin 1 receptor, MC1R; endoplasmic reticulum, ER; cyclic adenosine monophosphate, cAMP; phosphatidylinositol-3 kinase, PI3K; protein kinase B, AKT; melanocyte-inducing transcription factor, MITF; mitogen-activated protein kinase, MAPK; v-raf murine sarcoma viral oncogene homolog B1, BRAF; valine, V; glutamic acid, E; lysine, K; anti-cytotoxic T lymphocyte antigen-4, anti-CTLA-4; programmed cell death protein 1, PD-1; programmed cell death ligand 1, PDL1; melatonin, N-acetyl-5-methoxytryptamine; AFMK, N1-acetyl-N2-formyl-5-methoxykynuramine; 6-hydroxymelatonin, 6(OH)MEL; 5-methoxytryptamine 5-MT; 13-hydroxy octadecadienoic acid, 13-HODE; superoxide dismutase, SOD; glutathione GSH; glutathione peroxidase (GPx); reactive oxygen species, ROS; cyclin dependent kinase 1, CDK1; catalase, CAT; extracellular signal regulated protein kinase, ERK; 3-phosphoinositide-dependent protein kinase-1, PDK1; phosphatase and tensin homolog, PTEN; matrix metalloproteinase 1, MMP-1; epithelial-to-mesenchymal transition, EMT; nuclear factor kappa B, NF-kB; inhibitor of nuclear factor kappa-B kinase subunit beta, IKKβ; human telomerase reverse transcriptase, hTERT; inducible nitric oxide synthase, iNOS; epithelial cellular adhesion molecule, Epcam; proliferating cell nuclear antigen, PCNA.
